# Antimicrobial and Anti-Inflammatory Lingonberry Mouthwash—A Clinical Pilot Study in the Oral Cavity

**DOI:** 10.3390/microorganisms7090331

**Published:** 2019-09-08

**Authors:** Pirjo Pärnänen, Pirjo Nikula-Ijäs, Timo Sorsa

**Affiliations:** 1 Department of Oral and Maxillofacial Diseases, Helsinki University Hospital, University of Helsinki, 00014 Helsinki, Finland; 2 Faculty of Biological and Environmental Sciences, Molecular and Integrative Biosciences, The Department of Biochemistry and Biotechniques, University of Helsinki, 00014 Helsinki, Finland; 3 Department of Dental Medicine, Karolinska Institutet, Huddinge, 14104 Stockholm, Sweden

**Keywords:** *Vaccinium vitis-idaea*, fermentation, mouthwashes, *Streptococcus mutans*, *Candida*, *Lactobacillus*, point-of-care systems, anti-infective agents, dental plaque index

## Abstract

Fermented lingonberry juice was designed to be used as a mouthwash. Our aim was to study the antimicrobial and anti-inflammatory effects of the mouthwash in the oral cavity. A clinical study of 30 adult participants was performed. A total of 20 participants used 10 mL of the mouthwash twice daily for two weeks and 10 participants used 20 mL twice daily for one week. *Streptococcus mutans*, *Candida* and *Lactobacilli* were cultivated at the beginning, after the mouthwash period and after a washout period. At the same timepoints an additional oral mouthrinse was collected for chair-side/point-of-care (POC)-PerioSafe^®^/OraLyzer^®^ aMMP-8 quantitative on-line evaluation, and an oral clinical investigation was performed. Mean *Streptococcus mutans* and *Candida* counts, visible plaque index (VPI) and bleeding on probing (BOP) were reduced, and *Lactobacilli* counts increased during the lingonberry mouthwash period. The aMMP-8 mouthrinses showed reduced values in both test groups when compared to the startpoint. The mouthrinse aMMP-8 reduction correlated with the reductions in microbial counts, VPI and BOP. Based on the results, fermented lingonberry juice seems a promising aid in oral homecare, diminishing the microbial and related proinflammatory burden by balancing the oral microbial flora and gradually lowering the inflammatory load in the oral cavity.

## 1. Introduction

### 1.1. Background

Lingonberries (*Vaccinium vitis-idaea* L.) in the diet, like other berries, are thought to have beneficial health effects [1]. They contain phenolic substances [2,3] and several *in vitro* and *in vivo* studies show e.g., antioxidative [4,5], antimicrobial [6,7,8,9], anti-inflammatory [10,11], anticancerous [12,13] and improved hepatic function and glycemia [14] effects of lingonberries, but to our knowledge scarce clinical studies have been conducted on the effects to the oral cavity. Examples of an elevated inflammatory or infection risk include patients suffering from general conditions such as diabetes [15] or celiac disease [16] who may have oral manifestations, and local conditions such as periodontal disease [17]. The widespread use or misuse of antibiotics in dentistry [18] has increased microbial resistance problems and there is a demand for topical antimicrobial agents. Natural products are of great interest and wild berry-derived or even marine-derived compounds [19] have been studied increasingly.

Although lingonberries are considered healthy, they contain a lot of natural sugars even though they taste acidic. To utilize the phenolic substances safely in the oral environment, the sugar content of the cold-pressed lingonberry juice used in our study was lowered by a patented method [20]. An earlier study (60 patients) conducted by our group with concentrated unfermented lingonberry juice as a mouthwash showed lowered *S. mutans* and elevated *Lactobacillus* counts, but unexpectedly rising *Candida* counts and the study had to be interrupted (data not shown). Fermented lingonberry juice has been shown to inhibit certain *Candida glabrata* intracellular stress-related and energy metabolism enzyme expressions [21]. These findings may have an inhibitory effect on *C. glabrata* biofilm formation.

Neutrophil collagenase/collagenase-2 or matrix metalloproteinase (MMP)-8 is a collagenolytic and immunomodulatory proteolytic enzyme, and elevated levels of its active form (aMMP-8) reflect, precede and predict periodontal tissue destruction and inflammation [22,23,24]. Currently aMMP-8 can be measured accurately and on-line from the oral cavity by a quantitative chair-side point-of-care (POC) lateral flow reader-equipped oral mouthrinse immunotest (PerioSafe^®^/OraLyzer^®^) [22,23,24,25,26,27]. Microbial plaque accumulation to oral surfaces causes gingival bleeding on probing (BOP) reflecting inflammation, tooth caries and mucosal dysbiosis. Infection-induced increase in gingival inflammation (BOP) can also be detected and reflected by elevated aMMP-8 in mouthrinse [22,23,25,26,28].

### 1.2. Aims

Our aim was to test the clinical effect of fermented lingonberry juice as a mouthwash for balancing the oral microbial flora by reducing the amounts (CFU/mL, cultivation of patient oral saline rinse samples) of harmful microbes, e.g., *Streptococcus mutans* and *Candida*, aiding the growth of probiotic *Lactobacilli* in the oral cavity and lowering the inflammatory load (measured by aMMP-8 point-of-care mouthrinse method) caused by microbes. The relationship between oral harmful microbes and increased inflammation in the oral cavity has been debated. Our hypothesis is that fermented lingonberry used as a topical mouthwash could *in vivo* shift the microbial balance from a dysbiotic to a symbiotic direction [29,30], reduce inflammation and act as one tool aiding in oral homecare without adverse side-effects.

## 2. Results

*Candida* species determination showed *C. albicans* (24 patients), *C. parapsilosis* (3 patients), one *C. dubliniensis*, *C. guilliermondii* and *C. kefyr*.

**Figure 1:**
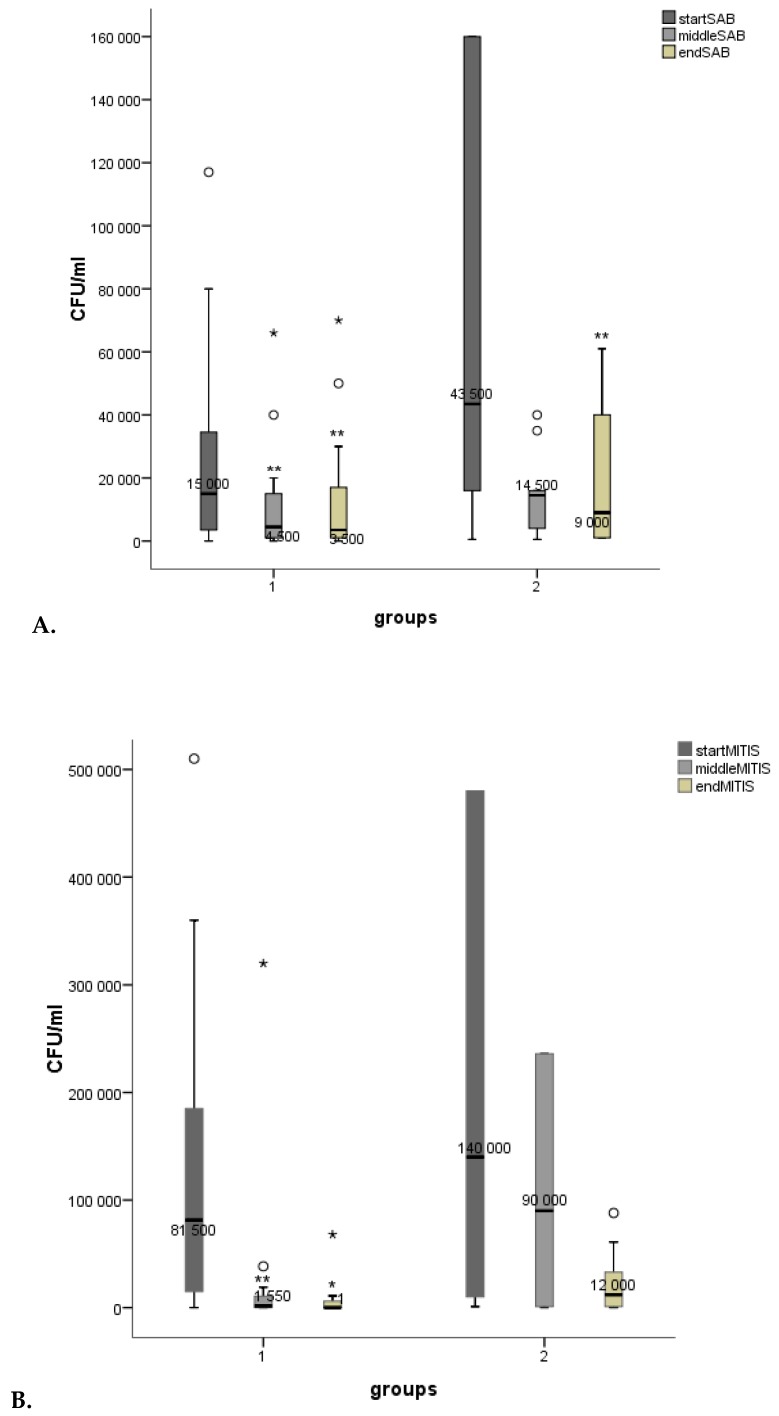

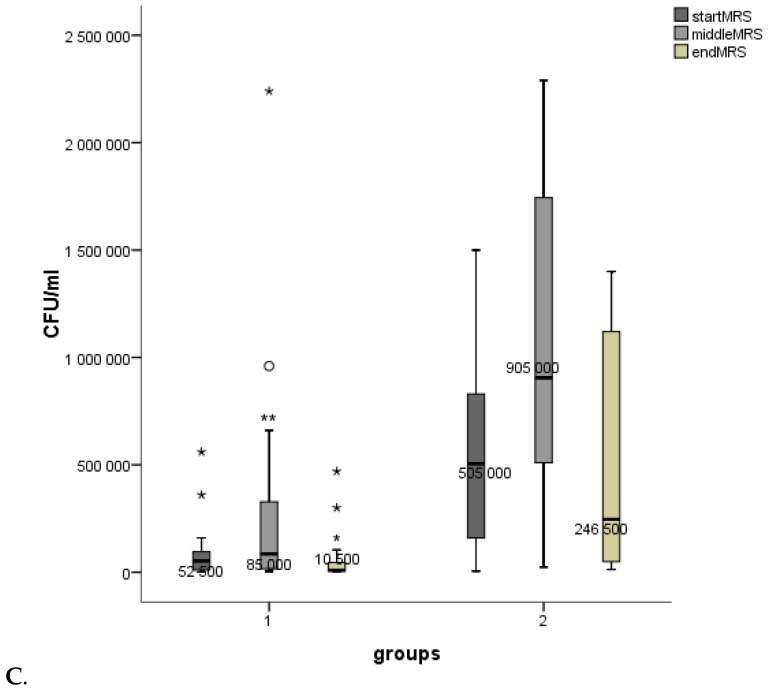
Boxplots for mean microbial counts (CFU/mL), (**A**). *Candida* (SAB), (**B**). *S. mutans* (MITIS) and (**C**). *Lactobacilli* (MRS). Measuring points for group 1 (start = 0 days, middle = 14 days, end = 28 days) and group 2 (start = 0 days, middle = 7 days, end = 14 days). *p* < 0.001***, *p* < 0.01**, *p* < 0.05* (smaller asterisks). Decreases/increases of microbial counts were compared pairwise: start/middle, start/end. Values more than three interquartile range (IQR) from the end of a box are labeled as extreme outliers (larger asterisks) and values more than 1.5 but less than 3 IQR’s from the end of a box are labeled (o) outliers.

The mean microbial counts from the timepoints are shown in Figure 1: A. *Candida*/Sabouraud dextrose agar (SAB), B. *S. mutans*/Mitis salivarius agar (MITIS), C. *Lactobacilli*/De Man, Rogosa and Sharpe agar (MRS). *Candida* and *S. mutans* counts diminished in both groups, (in group 1 significantly, *p* < 0.01). The *Lactobacillus* counts rose in both groups (in group 1 significantly, *p* < 0.01). 

**Figure 2:**
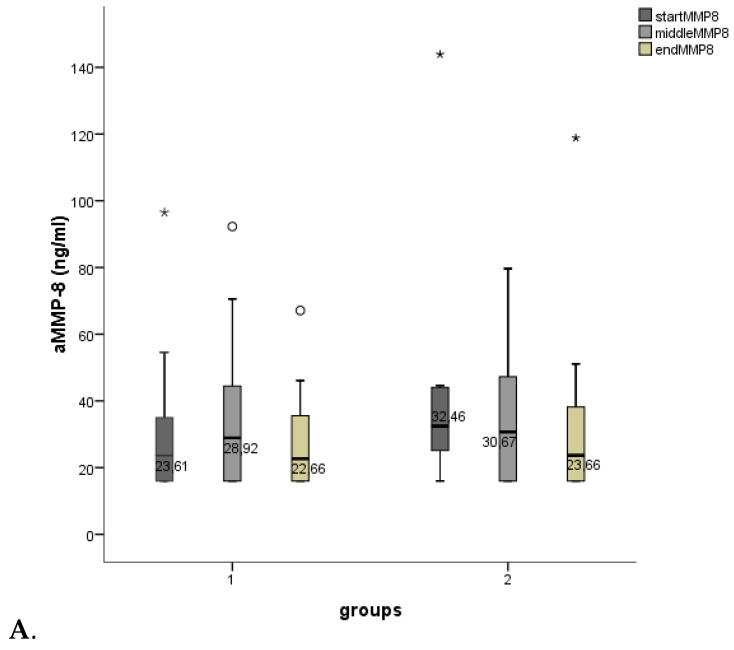

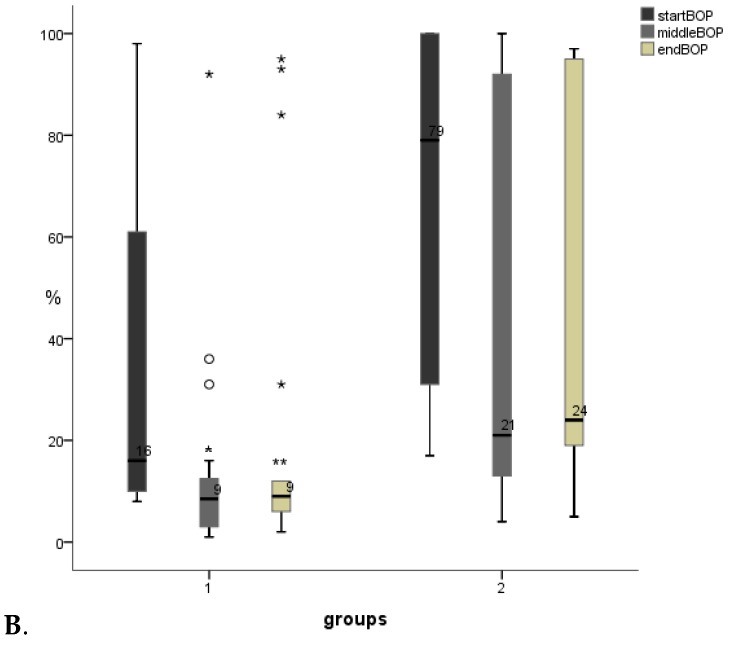
Boxplots for (**A**). aMMP-8 (ng/mL, PerioSafe^®^) and (**B**). BOP (%). Measuring points for group 1 (start = 0 days, middle = 14 days, end = 28 days) and group 2 (start = 0 days, middle = 7 days, end = 14 days). Decreases/increases of BOPs were compared pairwise: start/middle, start/end. *p* < 0.001***, *p* < 0.01**, *p* < 0.05* (smaller asterisks). Values more than three interquartile range (IQR) from the end of a box are labeled as extreme outliers (larger asterisks) and values more than 1.5 but less than 3 IQR’s from the end of a box are labeled (o) outliers.

The aMMP-8 oral rinse results are shown in Figure 2A. aMMP-8 values showed a lowering tendency in both groups during the study. BOP results are presented in Figure 2B. BOP values diminished in both groups (in group 1 significantly, *p* < 0.05).

**Figure 3:**
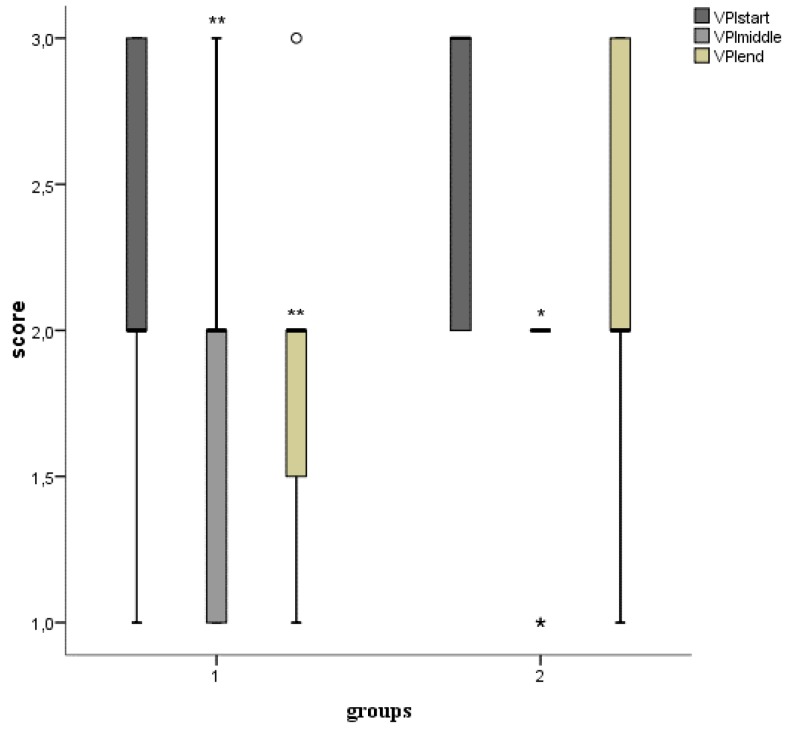
Boxplots for mean visible plaque index (VPI, scores 0 = no visible plaque3 = visible plaque on all tooth surfaces). Measuring points for group 1 (start = 0 days, middle = 14 days, end = 28 days) and group 2 (start = 0 days, middle = 7 days, end = 14 days). *p* < 0.001***, *p* < 0.01**, *p* < 0.05* (smaller asterisks). Decreases/increases of VPIs were compared pairwise: start/middle, start/end. Values more than three interquartile range (IQR) from the end of a box are labeled as extreme outliers (larger asterisks) and values more than 1.5 but less than 3 IQR’s from the end of a box are labeled (o) outliers.

The effect on visible plaque index (VPI) is shown in Figure 3. VPI diminished in both groups significantly (group 1: *p* < 0.01, group 2: *p* < 0.05). PPDs were not affected.

When groups were compared to each other, results show that BOPs were higher (start, *p* < 0.05; middle, *p* < 0.01; end, *p* < 0.05), *S. mutans* counts were higher (middle, *p* < 0,05) and *Lactobacilli* counts were higher (start, *p* < 0.01; middle, *p* < 0.01; end, *p* < 0.01) in group 2.

**Figure 4:**
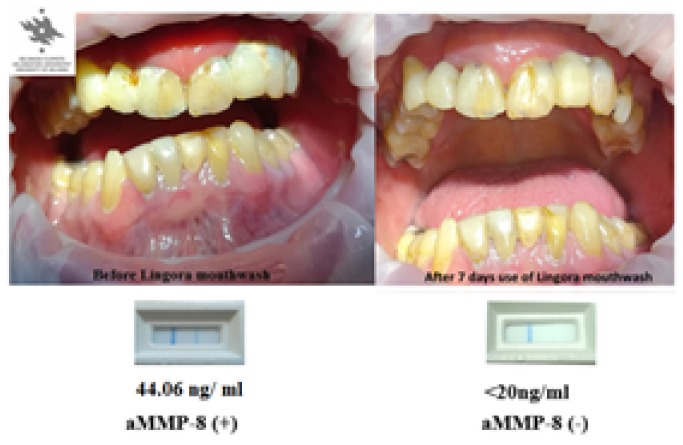
Photographs from one patient from group 2. (On the left before Lingora^®^ mouthwash; on the right 7 d after Lingora^®^ mouthwash). Below the photographs the PerioSafe^®^ chair-side test results (0 d, 7 d); two lines indicate positive (+, 44.06 ng/mL) and one line (−, < 20 ng/mL) negative test outcomes.

No alterations, as expected, in the caries status or pocket depths were noticed during the study periods. An example of the clinical beneficial effects of lingonberry mouthwash on one participant is shown in Figure 4. On clinical examination the participant’s gingiva showed reduced redness and swelling and diminished plaque on the tooth surfaces, that associated, was reflected and could be POC monitored the conversion of the positive aMMP-8 recordings’ conversion to negative aMMP-8 (< 20 ng/mL) recording.

**Figure 5:**
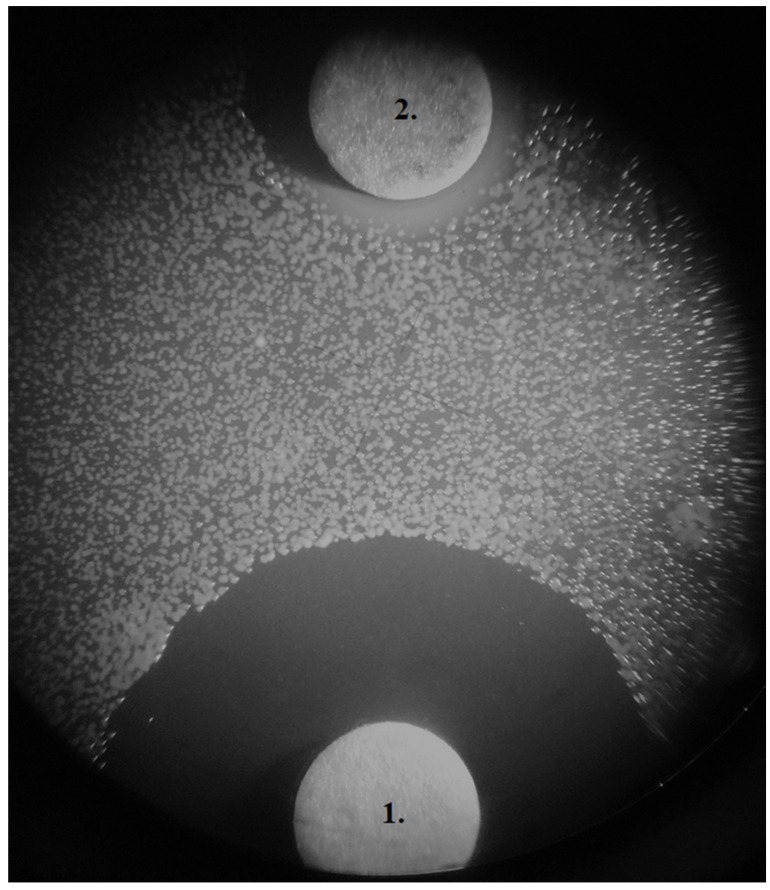
*In vitro* inhibitory zones on Brucella-agar. *Fusobacterium nucleatum* ATCC 25586 (100 µL McFarland 2); disc 1. 10 µL (0.2%) chlorhexidine (18 mm), disc 2. 100 µL Lingora^®^ (8 mm).

An example of *in vitro* inhibition of growth of *Fusobacterium nucleatum* by Lingora^®^ mouthwash compared to chlorhexidine is shown in Figure 5. Inhibitory zones of 18 mm (chlorhexidine) and 8 mm (Lingora^®^) were measured. Results from the other periodontopathogens tested showed similar results (not shown).

## 3. Discussion

*Candida* species distribution follows general findings [31,32]. In both groups the *S. mutans* and *Candida* counts diminished during/after the lingonberry mouthwash period. Additionally, the amount of visible plaque and bleeding on probing diminished. *Lactobacilli* counts showed a rising trend during the mouthwash period as expected, acting as beneficial balancing factor in the biofilm. Fermented lingonberry juice is rich in complex phenolic compounds which act as antioxidants [3,4]. Reactive oxygen species play a role in periodontal disease [33], and the inhibition of growth of periodontitis-related microbes would decrease the inflammatory burden of the tissue destruction and decrease the damage [22]. As an example proteases derived from certain *Candida* species and potent periodontopathogenic bacteria can convert latent proMMPs such as MMP-8 and MMP-9 into their active forms [34,35,36].

aMMP-8 values, which reflects the inflammation in the oral cavity, values showed a delayed drop towards the end of the investigation period (14 d/28 d). In fact, in group 2 the baseline values were higher, but overall trend was similar in both groups. The effect on the complex immune system seems to be a much slower event compared to the expression on oral microbes or clinical parameters, such as BOP and VPI. Fermented lingonberry juice seems to have a beneficial effect on these parameters, fading during the washout period. PPDs, as expected, were not affected during the two weeks lingonberry rinsing.

Chlorhexidine is widely used as an antimicrobial agent as mouthwash or gel. It is a potent antiseptic but has some adverse side effects such as pigmentation of teeth, cytotoxic effects, taste disturbance or alteration, oral mucosal soreness and irritation [37,38]. *In vitro* Brucella disc tests showed that Lingora^®^ mouthwash has an inhibitory effect of growth, albeit lower than chlorhexidine, of typical periodontopathogens. These findings need further investigations. Even though the inhibitory effect of the lingonberry mouthwash was lower compared to chlorhexidine, the advantage is that it can be used daily, contrary to chlorhexidine which is recommended to be used for a limited time. One other advantage of the lingonberry mouthwash is that it allows the patient’s own *Lactobacillus* flora to persist and even expand, cutting space from opportunistic oral pathogens to grow.

Overall, fermented lingonberry juice seems a promising and safe aid in oral homecare in reducing harmful microbes, plaque, gingival bleeding and has an anti-inflammatory/antioxidant effect through lowering aMMP-8 load in the oral cavity. This is to our knowledge the first study comparing microbial counts to mouthrinse aMMP-8 levels detected by chair-side/point-of-care technology that is available to dental and medical professionals. Further studies are still needed to corroborate these results.

## 4. Materials and Methods

Fermented lingonberry juice from the genus *Vaccinium vitis-idaea* L. (Lingora^®^, Vantaa, Finland) was used as a lingonberry mouthwash. It is concentrated, containing appr. 1/10 of naturally occurring sugars and has no additives. A total of 30 adult patients from a general dentist’s office in Helsinki, Finland (M/F ratio was 21/9, mean age 67 years) were divided into two groups to evaluate the effect of dosage and duration of the mouthwash period on the parameters measured. Our pilot study has received approval from the ethical committee of Stockholm Community, Sweden (2016-08-24/2016/1:8 and 2016-1-24) and the Helsinki University Central Hospital, Finland (360/13/03/00/13 and 51/13/02/2009). A total of 20 participants used 10 mL of the lingonberry mouthwash (Lingora^®^) twice daily (30 s) for two weeks (group 1) and 10 participants used 20 mL twice daily (30 s) for one week (group 2). A total of 10 mL of saline was rinsed for 30 s and rinse was used for microbial cultivation.

*Streptococcus mutans*, *Candida* and *Lactobacilli* were cultivated (0 d, 7 d, 14 d, 28 d) from group 1 and (0 d, 3 d, 7 d, 14 d) from group 2. Streptococcal counts were calculated from Mitis salivarius (MITIS) agar (Merck, Darmstadt, Germany) (48 h, verified with phase-contrast microscopy for typical *S. mutans* colony morphology), *Candida* from Sabouraud dextrose (SAB) agar (LabM, Bury, Great Britain) (24 h) and *Lactobacillus* counts (48 h) from De Man, Rogosa and Sharpe (MRS) agar (Merck, Darmstadt, Germany) by the dilution method. *Candida* colonies were cultivated on chromagar and species determination was conducted with API 20 C AUX (bioMerieux, Lyon, France).

At the same timepoints, an additional oral mouthrinse was collected and analyzed, according to the manufacturer’s instructions, for aMMP-8 levels expressed as aMMP-8 ng/mL (PerioSafe^®^/OraLyzer^®^, Dentognostics, Jena, Germany) by a quantitative on-line evaluation [24,25,26,27,28]. 

An oral clinical investigation was performed at each timepoint, including periodontal pocket depths (PPDs), carious lesions, visible plaque (scale from VPI (0) = no plaque up to VPI (3) = plaque on all tooth surfaces) and bleeding on probing (BOP) [25,26]. Additionally, smoking habits, brushing frequency, illnesses, and medication were recorded. The patients’ homecare or eating habits were continued throughout the examination period as usual.

After the study the patients were treated by ultrasonic depuration and scaling/root planing. 

### 4.1. Statistical Analysis

The effects of the lingonberry mouthwash were compared with paired sample t-tests (confidence interval (CI) 95%) within groups (decreases/increases of the parameters within each group were compared pairwise: start/middle, start/end) and with one-way analysis of variance (ANOVA) between groups. Skewness was log10 transformed in all variables except VPI prior to analysis. SPSS 24 (IBM, NY, USA) was used for data analyses.

### 4.2. Additional Preliminary In Vitro Periodontopathogen tests

Inhibition of growth with Lingora^®^ of typical periodontopathogens *Aggregatibacter actinomycetemcomitans* (*A.a*) ATCC 29523 (serotype a) and ATCC 43718 (serotype b), *Porphyromonas gingivalis* (*P.g*.) ATCC 33277 (serotype a) and W50, and *Fusobacterium nucleatum* (*F.n.*) ATCC 25586 were tested. A total of 100 µL of Lingora^®^ mouthwash was applied onto a 6 mm paper disc on Brucella agar and 10 µL of 0.2% chlorhexidine was used as a control. The plates were incubated at 37 °C (*A.a.* microaerophilic, *P.g.*, and *F.n.* anaerobic conditions) until sufficient growth.
